# An integrated national mortality surveillance system for death registration and mortality surveillance, China

**DOI:** 10.2471/BLT.15.153148

**Published:** 2015-10-28

**Authors:** Shiwei Liu, Xiaoling Wu, Alan D Lopez, Lijun Wang, Yue Cai, Andrew Page, Peng Yin, Yunning Liu, Yichong Li, Jiangmei Liu, Jinling You, Maigeng Zhou

**Affiliations:** a National Center for Chronic and Non-communicable Disease Control and Prevention, Chinese Center for Disease Control and Prevention, 27 Nanwei Road, Xicheng District, Beijing, 100050, China.; b Center for Health Statistics and Information, National Health and Family Planning Commission, Beijing, China.; c School of Population and Global Health, University of Melbourne, Melbourne, Australia.; d School of Science and Health, University of Western Sydney, Sydney, Australia.

## Abstract

In China, sample-based mortality surveillance systems, such as the Chinese Center for Disease Control and Prevention’s disease surveillance points system and the Ministry of Health’s vital registration system, have been used for decades to provide nationally representative data on health status for health-care decision-making and performance evaluation. However, neither system provided representative mortality and cause-of-death data at the provincial level to inform regional health service needs and policy priorities. Moreover, the systems overlapped to a considerable extent, thereby entailing a duplication of effort. In 2013, the Chinese Government combined these two systems into an integrated national mortality surveillance system to provide a provincially representative picture of total and cause-specific mortality and to accelerate the development of a comprehensive vital registration and mortality surveillance system for the whole country. This new system increased the surveillance population from 6 to 24% of the Chinese population. The number of surveillance points, each of which covered a district or county, increased from 161 to 605. To ensure representativeness at the provincial level, the 605 surveillance points were selected to cover China’s 31 provinces using an iterative method involving multistage stratification that took into account the sociodemographic characteristics of the population. This paper describes the development and operation of the new national mortality surveillance system, which is expected to yield representative provincial estimates of mortality in China for the first time.

## Résumé

En Chine, des systèmes de surveillance de la mortalité fondés sur des échantillons (comme le système à points de surveillance des maladies du Centre chinois pour le contrôle et la prévention des maladies et le système d'enregistrement des données d'état civil du ministère de la Santé) sont utilisés depuis plusieurs dizaines d'années pour obtenir des données représentatives à l'échelle nationale de la situation sanitaire, en vue d'éclairer les processus décisionnels en matière de santé et d'évaluer les résultats. Or, aucun de ces systèmes traditionnellement utilisés ne proposait de données représentatives sur la mortalité et les causes de décès à l'échelle provinciale, pourtant nécessaires pour correctement définir les priorités et les besoins régionaux en matière de politiques de santé. Par ailleurs, ces systèmes étaient largement redondants entre eux, ce qui impliquait donc une duplication inutile des efforts. En 2013, le gouvernement chinois a fusionné ces deux systèmes dans un système national intégré de surveillance de la mortalité afin d'obtenir une image représentative à l'échelle provinciale de la mortalité totale et de la mortalité par cause et d'accélérer la création d'un système exhaustif d'enregistrement des données d'état civil et de surveillance de la mortalité pour tout le pays. Ce nouveau système a permis d'augmenter la couverture de la surveillance (de 6% de la population chinoise couverte auparavant à 24%). Le nombre de points de surveillance (chacun couvrant un district ou un comté) est passé de 161 à 605. Pour garantir une bonne représentativité à l'échelle provinciale, les 605 points de surveillance ont été sélectionnés de manière à couvrir les 31 provinces chinoises à l'aide d'une méthode itérative impliquant une stratification à plusieurs degrés qui a tenu compte des caractéristiques sociodémographiques de la population. Cet article décrit l'élaboration et le fonctionnement de ce nouveau système national de surveillance de la mortalité, qui devrait permettre d'obtenir pour la première fois des estimations représentatives à l'échelle provinciale de la mortalité en Chine.

## Resumen

En China, los sistemas de vigilancia de la mortalidad basados en muestras, tales como el sistema de puntos de vigilancia de las enfermedades del Centro de Prevención y Control de Enfermedades de China y el sistema de registro civil del Ministerio de Salud, se han utilizado durante décadas para proporcionar datos nacionalmente representativos del estado de salud para tomar decisiones médicas y evaluaciones de rendimiento. Sin embargo, ningún sistema ofrecía datos representativos en cuanto a defunciones y las causas de las defunciones a un nivel provincial con el objetivo de informar de las necesidades de servicios sanitarios regionales y las prioridades de la política. Asimismo, los sistemas se solapaban hasta un punto considerable, lo que suponía una duplicación de los esfuerzos. En 2013, el gobierno chino combinó estos dos sistemas en un sistema nacional integrado de vigilancia de la mortalidad para proporcionar una imagen provincialmente representativa de la mortalidad total y de la mortalidad por causas específicas y para acelerar el desarrollo de un registro civil completo y un sistema de vigilancia de la mortalidad para todo el país. Este nuevo sistema aumentó la población de vigilancia de un 6 a un 24% de la población china. El número de puntos de vigilancia, donde cada uno cubría un distrito o condado, subió de 161 a 605. Con el objetivo de garantizar una representación a nivel provincial, los 605 puntos de vigilancia se seleccionaron para cubrir las 31 provincias de China mediante la utilización de un método iterativo que consistía en una estratificación de etapas múltiples que tenía en cuenta las características sociodemográficas de la población. Este artículo describe el desarrollo y funcionamiento del nuevo sistema nacional de vigilancia de la mortalidad, el cual se espera que aumente las estimaciones provinciales representativas de mortalidad en China por primera vez.

## ملخص

لقد تم استخدام أنظمة مراقبة الوفيات المستندة إلى العينات، مثل نظام نقاط مراقبة الأمراض التابع للمركز الصيني لمكافحة الأمراض والوقاية منها، ونظام السجلات المدنية الخاصة بوزارة الصحة هناك، لعدة قرون لتوفير بيانات تمثيلية وطنية حول الحالة الصحية لأغراض اتخاذ القرارات المتعلقة بالرعاية الصحية، وتقييم الأداء. إلا أن أياً من هذه الأنظمة لم يقدم بيانات تمثيلية حول الوفيات أو أسباب حدوثها على مستوى المقاطعات حتى يوضح احتياجات الخدمة الصحية الإقليمية وأولويات السياسة. وعلاوة على ذلك، حدث تداخل بين الأنظمة إلى درجة ملموسة، مما يؤدي بدوره لمضاعفة الجهود. وقامت الحكومة الصينية في عام 2013 بدمج هذين النظامين في نظام وطني واحد متكامل لمراقبة الوفيات، بهدف تقديم صورة تمثيلية للوفيات بشكل عام ولأسباب الوفيات على مستوى المقاطعات، وللتعجيل بتطوير سجلات مدنية شاملة ونظام مراقبة شامل للوفيات لجميع أنحاء الدولة. وقد أدى هذا النظام الجديد لاتساع مدى المراقبة من 6% من الشريحة السكانية إلى 24%. وقد زاد عدد نقاط المراقبة، والتي تغطي كل منها منطقة أو مقاطعة، من 161 إلى 605 نقطة. وللتحقق من مصداقية تمثيل البيانات على مستوى المقاطعات، فقد تم اختيار الـ 605 نقطة مراقبة بحيث تغطي 31 مقاطعة في الصين، باستخدام طريقة تكرارية تنطوي على تصنيف متعدد المراحل، وتضع في الحسبان الخصائص الاجتماعية الديموغرافية للسكان. تشرح هذه الورقة كيفية تطوير وتشغيل نظام مراقبة الوفيات الوطني الجديد، والذي من المتوقع أن يقدم تقديرات تمثيلية للوفيات على مستوى المقاطعات لأول مرة في الصين.

## 摘要

在中国,基于样本的死亡率监测系统,如中国疾病预防控制中心的疾病监测点系统和卫生部的生命登记系统,几十年一直用于提供有关健康状态的全国代表性数据,以进行准确的医疗保健决策和绩效评估。然而,这两个系统都无法在死亡率和死因方面提供省级代表性数据,以告知地区卫生服务需求和政策优先事项。此外,这两个系统在相当大的程度上互相重叠,因此导致重复劳动。2013 年,中国政府将这两个系统纳入全国死亡率监测综合系统,在总体死亡率和死因特定死亡率方面提供省级代表性现象,并加速全国生命登记和死亡率监测综合系统的发展。这个新系统将监测人口从中国总人口的 6% 提升至 24%。涵盖区或县的各监控点数量从 161 个增至 605 个。为了确保省级的代表性,使用迭代法,其中包括基于人口社会人口学特征的多层分级法,选择 605 个监控点,覆盖中国 31 个省。本文介绍了全国新型死亡率监测系统的形成和运行,预计将首次得出中国死亡率的省级代表性估计值。

## Резюме

В Китае системы выборочного контроля за смертностью, такие как система пунктов контроля заболеваний Китайского центра по профилактике болезней и борьбе с ними и система регистрации естественного движения населения Министерства здравоохранения, используются на протяжении десятилетий для получения национально-репрезентативных данных о состоянии здоровья, на основе которых принимаются решения и оцениваются результаты в сфере здравоохранения. Однако ни одна из этих систем не позволяла получить репрезентативные данные о смертности и причине смерти на уровне провинций, которые позволили бы определить потребности и приоритеты политики региональных служб здравоохранения. Кроме того, сферы деятельности двух систем во многом пересекались, вследствие чего происходило дублирование работы. В 2013 году Правительство Китая объединило эти две системы в единую национальную систему контроля за смертностью для получения репрезентативной картины общей смертности с распределением по провинциям и смертности с разбивкой по причинам смерти. Это было сделано также для ускорения разработки всеобъемлющей системы регистрации естественного движения населения и контроля за смертностью для всей страны. Благодаря этой новой системе количество жителей, входящих в сферу контроля, увеличилось с 6 до 24% от всего населения Китая. Количество пунктов контроля, каждый из которых привязан к округу или району, было увеличено с 161 до 605. В целях обеспечения репрезентативности на уровне провинций 605 пунктов контроля были отобраны для курирования 31 провинции Китая с помощью метода итерации, включающего многоэтапную стратификацию, которая учитывала социально-демографические особенности населения. В данной статье описывается развитие и работа новой национальной системы контроля за смертностью, которая, как ожидается, впервые позволит осуществить репрезентативную оценку смертности в Китае на уровне провинций.

## Introduction

Reliable and timely information on cause-specific mortality is fundamental for informing the development, implementation and evaluation of health policy.^1^^,^^2^ China has yet to establish a complete vital registration system for its 1.3 billion population. To date, the essential data on the causes of death in China used for decision-making and performance evaluation have come from sample-based mortality surveillance systems, including the nationally representative disease surveillance points system of the Chinese Center for Disease Control and Prevention (CDC) and the vital registration system of the Chinese Ministry of Health.^3^

The disease surveillance points system was established in 1978 with a pilot study at two surveillance points in Beijing.^3^ By 1990, the number of points had increased to 145 and the population covered was approximately 10 million.^3^^,^^4^ In 2004, the system was expanded again to include 161 points and population coverage increased to 73 million. The sampling strategy and the characteristics of this system have been described in detail elsewhere along with the quality control measures and the procedures for collecting data, coding the cause of death and determining the underlying cause of death.^3^^–^^5^ For deaths in hospital, doctors certified the cause of death and trained coders determined the underlying cause of death by applying the rules of the International Classification of Diseases.^6^ For hospitals without the capacity to determine or code the underlying cause of death, these functions were carried out by the county or district CDC. For deaths occurring outside hospital, village health workers or township or community hospital staff did a verbal autopsy from which doctors in these hospitals determined the underlying cause of death. Since 2008 information on individual deaths in all population catchment areas has been reported in real time using an Internet-based reporting system.^7^ In this system, information on each death is systematically validated by local – including county, prefecture and provincial level – Centers for Disease Control and Prevention, which also check the completeness, coding and internal logic of the items reported on death certificates. Causes-of-death are subsequently reported to the national CDC, where data are consolidated.

The Chinese vital registration system was established in the 1950s to collect mortality data in 13 cities. By 2000, the population under surveillance was around 110 million and the system covered 15 large cities, 21 medium-sized or small cities and 90 counties drawn from 15 provinces and municipalities.^8^ By 2012, the system had expanded to include 319 sites (138 counties and 181 districts) in 22 provinces covering about 230 million people, mostly in eastern and central areas of the country. Forty-two counties in the vital registration system overlapped with population catchments in the disease surveillance points system. The collection of cause-of-death data in the vital registration system was similar to the disease surveillance points system. Data on deaths were compiled according to predetermined aggregation principles and reported monthly by electronic file transfer to the Center for Health Statistics and Information of the National Health and Family Planning Commission (previously the Ministry of Health). Quality control meetings were held annually and regular training was carried out to ensure data quality.

Together, the disease surveillance points and vital registration systems provided a nationally representative picture of mortality in China.^9^ The vital registration system, while not representative, was able to give more accurate estimates of the proportion of deaths due to specific causes and larger sample of deaths than the disease surveillance points. The disease surveillance points system reflected total mortality, the broad cause-of-death distribution and the geographic distribution of deaths more accurately, because the sampling strategy employed ensured a nationally representative sample.^10^ However, neither system was able to provide representative data on mortality or the causes of death at the provincial level. Differences between the two systems and their development are described more fully in Table 1 and Table 2.

**Table 1:** Vital registration and disease surveillance points mortality surveillance systems, China, 1950–2013

Mortality surveillance system	Establishment	Development	Sampling method	Sites covered	Population coverage	Representativeness	Data reporting
**Vital registration system**	During the 1950s in 13 cities, including Beijing, Shanghai and Nanjing	By 1985, 28 large and medium-sized cities and 70 counties were covered; by 2000, 36 cities (i.e. municipalities and prefecture-level cities, which included many districts) and 90 counties in 15 provinces were covered.	Voluntary	Cities (including counties and districts) and counties	By 1985, 60 million; by 2000, 110 million	Mostly cities and eastern rural areas of the country	Local CDCs made monthly reports by electronic file transfer to the CHSI of the NHFPC
		By 2012, the system included 319 surveillance points^a^ in 22 provinces	Voluntary	Counties and districts	By 2012, 230 million	Mostly eastern and central areas of the country	Local CDCs made monthly reports by electronic file transfer to the CHSI of the NHFPC
**Disease surveillance points system**	In 1978, in Dongcheng district and Tong county, Beijing	By 1989, 71 surveillance points^a^ across 29 provinces were covered	Voluntary	ND	ND	Mostly large cities and more wealthy rural areas	Local CDCs made monthly reports by electronic file transfer to the national CDC
		In 1990, 145 surveillance points^a^ across 31 provinces were covered	Multistage, stratified, cluster sampling (sampling probability proportional to population size)	1 or 2 townships in each county or 1 or 2 subdistricts in each district for each surveillance point	10 million (approximately 1% of the total Chinese population)	Both nationally and regionally (i.e. eastern, central and western; urban and rural) representative	Local CDCs made monthly reports by electronic file transfer to the national CDC
		In 2004, 161 surveillance points^a^ across 31 provinces were covered	Multistage stratification, selection, evaluation and adjustment	The whole population covered by each surveillance point	73 million (approximately 6% of the total Chinese population)	Both nationally and regionally (i.e. eastern, central and western; urban and rural) representative	Since 2008, hospitals and local CDCs have reported to the national CDC using a real-time Internet-based system

CDC: Center for Disease Control and Prevention; CHSI: Center for Health Statistics and Information; ND: not determined; NHFPC: National Health and Family Planning Commission (previously the Ministry of Health).

^a^ Each surveillance point corresponded to one county or district.

**Table 2:** Reporting cause-of-death through vital registration and disease surveillance points systems, China, 1950–2013

Period^a^, by place of death	Vital registration system					Disease surveillance points system			
	Death information collection	Death certificate	Coding cause of death	Determining the underlying cause of death		Death information collection	Death certificate	Coding cause of death	Determining the underlying cause of death
In earlier years, in hospital	Family members reported to local vital registration offices in hospitals, hospitals reported to local CDCs and local CDCs prepared summaries	Staff in the hospital’s vital registration office completed the certificate using information from family members and any available medical records and documents	Coding was done by staff in local CDCs; ICD-10 classification used since 2002	Determined by staff in local CDCs		Staff in the hospital’s disease prevention unit collected death certificates and reported to the local CDC, which provided summaries	Clinical doctors in the hospital completed the certificates	Initially, coding was done by staff in the national CDC (previously the Chinese Academy of Preventive Medicine); then there was a gradual transition to the procedures used in recent years. ICD-10 classification used since 2004	Initially determined by staff in the national CDC (previously the Chinese Academy of Preventive Medicine); then there was a gradual transition to the procedures used in recent years
In recent years, in hospital	Staff in the hospital’s disease prevention unit collected death certificates and reported to the local CDC, which provided summaries	Clinical doctors in the hospital completed the certificates	Coding was done either by: doctors in the hospital’s medical records unit or by staff in the hospital’s disease prevention unit, and local CDC staff checked the coding; or local CDC staff when the local hospital did not have the capacity. ICD-10 classification used since 2002	Determined either by doctors in the hospital’s medical records unit or by staff in the hospital’s disease prevention unit, and checked by local CDC staff; or by local CDC staff when the local hospital did not have the capacity		Staff in the hospital’s disease prevention unit collected death certificates and entered the details onto a real-time, Internet-based system; local CDC staff checked the information	Clinical doctors in hospitals completed the certificates	Coding was mostly done by local CDC staff, but also by doctors in hospitals’ medical records units or staff in hospitals’ disease prevention units, and checked by local CDC staff. ICD-10 classification used since 2004	Mostly determined by local CDC staff, but also by doctors in hospitals’ medical records units or staff in hospitals’ disease prevention units, and checked by local CDC staff
In earlier years, outside hospital	Family members reported to local vital registration offices in local hospitals, hospitals reported to local CDCs and local CDCs prepared summaries	Staff in local hospitals’ vital registration offices completed the certificates using information from family members and any available medical records and documents	Coding was performed by staff in local CDCs; ICD-10 classification used since 2002	Determined by staff in local CDCs		Village health workers and disease prevention unit staff in township hospitals in rural areas and disease prevention unit staff in CHs in urban areas collected information using household surveys; the information was checked with bodies such as the local police, the civil affairs department and the maternal and child department and then reported to the local CDC, which prepared summaries	Staff in local hospitals’ disease prevention units completed the certificates using information from family members and any available medical records and documents	Coding was performed by staff in the national CDC (previously the Chinese Academy of Preventive Medicine); ICD-10 classification used since 2004	Determined by staff in the national CDC (previously the Chinese Academy of Preventive Medicine)
In recent years, outside hospital	Village health workers in rural areas and disease prevention unit staff in CHs in urban areas collected information using household surveys; the information was checked with bodies such as the local police, the civil affairs department and the maternal and child department and then reported to the local CDC, which prepared summaries	Certificates were completed by clinical doctors in charge of emergency treatment or by disease prevention unit staff in charge of household surveys or of checking field reports	Coding was done either by the local hospital’s disease prevention unit staff, and checked by local CDC staff; or local CDC staff when the local hospital did not have the capacity. ICD-10 classification used since 2002	Determined by either: (i) the local hospital’s disease prevention unit staff, and checked by local CDC staff; or (ii) local CDC staff when the local hospital did not have the capacity		Village health workers and disease prevention unit staff in township hospitals in rural areas and disease prevention unit staff in CHs in urban areas collected information using household surveys; the information was checked with agencies such as the local police, the civil affairs department and the maternal and child department and then entered onto a real-time, Internet-based system; local CDC staff checked the information	Certificates were completed by clinical doctors in charge of emergency treatment or by disease prevention unit staff in charge of household surveys or of checking field reports	Coding mostly done by local CDC staff, but also by staff in hospitals’ disease prevention units, with local CDC staff checking the information; ICD-10 classification used since 2004	Mostly determined by local CDC staff, but also by staff in hospitals’ disease prevention units, and local CDC staff checked the information

CDC: Center for Disease Control and Prevention; CH: community hospital or community health centre or station; ICD-10: *International statistical classification of diseases and related health problems,* 10^th^ revision.

^a^ Exact time cannot be given since this was a gradual process and different sites have different starting time points.

Note: Local CDC refers to county and district levels.

In 2013, the National Health and Family Planning Commission combined the vital registration system and disease surveillance points system to create an integrated national mortality surveillance system. The goals were to integrate and rationalize the health resources expended on these systems and to accelerate the development of a complete vital registration and mortality surveillance system covering the entire population of China. Initially, the national mortality surveillance system covered a population of 323.8 million (24.3% of the total population of the country) and comprised 605 surveillance points, with each point covering an entire county or district. This paper describes the development and operation of this new mortality surveillance system.

## Development of a new system

**Fig. 1:**
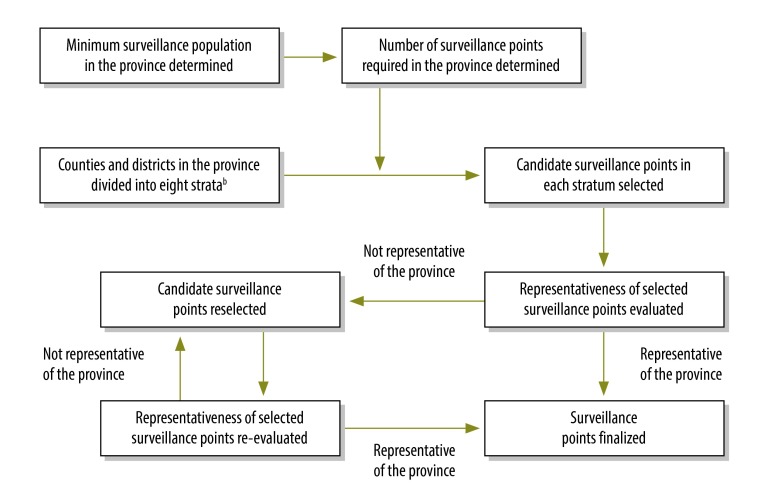
Selection^a^ of points in the national mortality surveillance system in each province, China, 2013

^a^ An iterative process was used to determine whether the population covered by the combination of selected surveillance points in a province was representative of the population of the whole province. If the combination was not representative of the whole province, different points were selected within each stratum until it was representative; however, the number of points in each stratum was not changed.

^b^ Counties and districts were divided into eight strata according to their degree of urbanization, population size and crude mortality rate.

The national mortality surveillance system was established using the same general principles applied in developing the disease surveillance points system.^3^^–^^5^ First, the National Health and Family Planning Commission determined that the surveillance population should be not less than 5 million in any province that had a population greater than 10 million and was economically well developed; for other provinces, the population sample had to be at least 20% of the total population. These criteria were used to establish the number of surveillance points required in each province. Second, we divided all counties and districts in each province into eight strata according to their degree of urbanization, population size and the crude mortality rate (total number of deaths per 1000 people per year). Third, we selected counties and districts in each stratum as candidate surveillance points for each province in accordance with the number of surveillance points required. We then determined how representative the candidate surveillance points were of the whole province using data from the 2010 census.^11^ The final surveillance points for each province were selected using an iterative process that ensured the combination of points was representative of the population of the province (Fig. 1).

### Surveillance population

**Fig. 2:**
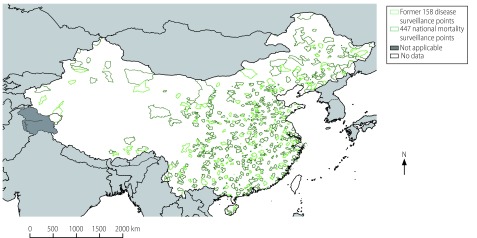
Surveillance points, national mortality surveillance system, China, 2013

Note: Dark green lines represent 447 counties with national mortality surveillance points; light green lines represent 158 counties which also have disease surveillance points. The map was produced using QGIS version 2.8.3 (QGIS Development Team).

Source: Map data for China from the Natural Resources and Geospatial Base Information Database (National Development and Reform Commission, China). Map data for neighbouring countries from WHO.

The final size of the target surveillance population in each province was based not only on the two criteria used by the National Health and Family Planning Commission as described above but also on decisions made by the national CDC in consultation with provincial CDCs, which took into consideration differences between provinces in population and in the capability and training of health-care staff and their ability to implement the new surveillance system. For example, the surveillance population chosen for Beijing was large because the city has a good infrastructure, a well-trained workforce and a large population. In contrast, the surveillance population in western provinces was smaller, partly because the local capacity and resources available for carrying out reliable mortality surveillance were limited. Following detailed consideration of the average population of all counties and districts and after consultation with health authorities in each province, we determined that the national mortality surveillance system required a total of 605 surveillance points – the 605 counties and districts covered by these points comprised 21.1% of all counties and districts in China (Table 3; Fig. 2).

**Table 3:** Surveillance points in the national mortality surveillance system, China, 2013

Province	No. of counties and districts^a^	No. of surveillance points^b^ (% of counties and districts)
Anhui	105	24 (22.9)
Beijing	18	7 (38.9)
Chongqing	40	11 (27.5)
Fujian	84	20 (23.8)
Gansu	87	20 (23.0)
Guangdong	123	28 (22.8)
Guangxi	110	21 (19.1)
Guizhou	88	20 (22.7)
Hainan	24	8 (33.3)
Hebei	172	30 (17.4)
Heilongjiang	132	27 (20.5)
Henan	159	36 (22.6)
Hubei	103	22 (21.4)
Hunan	122	28 (23.0)
Inner Mongolia	101	20 (19.8)
Jiangsu	106	27 (25.5)
Jiangxi	99	20 (20.2)
Jilin	60	15 (25.0)
Liaoning	100	22 (22.0)
Ningxia	22	10 (45.5)
Qinghai	46	10 (21.7)
Shaanxi	107	13 (12.1)
Shandong	140	31 (22.1)
Shanghai	18	7 (38.9)
Shanxi	119	20 (16.8)
Sichuan	181	31 (17.1)
Tianjin	16	7 (43.8)
Tibet	73	8 (11.0)
Xinjiang	98	15 (15.3)
Yunnan	129	25 (19.4)
Zhejiang	90	22 (24.4)
**Total**	**2872**	**605 (21.1)**

^a^ The number of counties and districts in each province was the same as the number used in the 2010 national census.^11^

^b^ Each surveillance point covered an entire county or district.

### Stratification

**Fig. 3:**
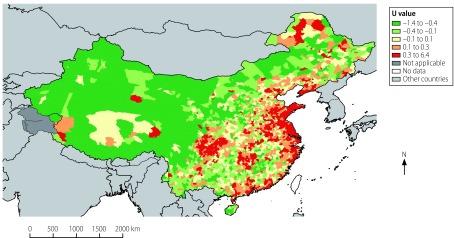
Urbanization, population size and mortality rate, China, 2013

Notes: The county or district representation was calculated as follows: first each *U*-value for the urbanization index, population size and mortality rate was obtained by using the equation *U* =  (*x*−*μ*)/*σ*, where x is the observed value of the particular index in the country or district, *μ* is the mean value of the index in all counties and districts in the province and *σ* is the standard deviation. We then calculated the mean *U*-value for the three indices for each county or districts. The map was produced using QGIS version 2.8.3 (QGIS Development Team).

Source: Map data for China from the Natural Resources and Geospatial Base Information Database (National Development and Reform Commission, China). Map data for neighbouring countries from WHO.

In the previous two adjustments to the disease surveillance points system in 1990 and 2004, urban areas (i.e. districts) and rural areas (i.e. counties and county-level cities) were used as the primary units for stratification and the urban-to-rural population ratio was also taken into account.^3^^–^^5^ However, with the rapid socioeconomic development of the last decades, this ratio is no longer appropriate for defining a county or county-level city as a rural area or a district as an urban area. In addition, per-capita gross domestic product (GDP) was used only in the further stratification of rural areas because there was a lack of data on how urban per capita GDP varied by district.^3^^–^^5^ Given the incompleteness of these data and the potential positive correlation between urbanization and per capita GDP, we decided to use the urbanization index (i.e. the fraction of the population residing in an urban area) as a stratifying index instead of the urban-to-rural population distribution or per-capita GDP. Following consultations with experts, population size was retained as an important stratifying index in the selection of surveillance points for the National Mortality Surveillance System, as was the crude mortality rate. These three stratifying indices were used as descriptors for each surveillance point and were calculated for each province. To obtain a graphical illustration of the characteristics in each county or district based on the three stratification indices, we first calculated the representation (*U*) for each index as follows: *U* =  (*x*−*μ*)/*σ*, where *x* is the observed value of the particular index in the country or district, *μ* is the mean value of the index in all counties and districts in the province and *σ* is the standard deviation. We then calculated the mean *U*-value for the three indices for each county or district (Fig. 3).

**Fig. 4:**
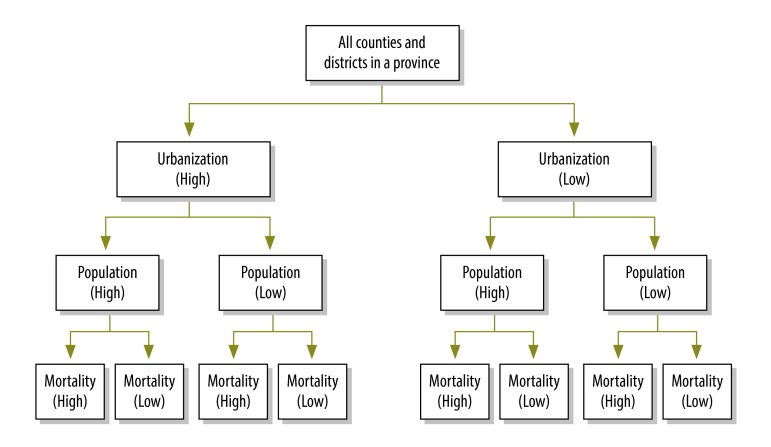
Stratification^a^ of counties and districts in each province for the development of the national mortality surveillance system, China

^a^ Counties and districts were divided sequentially into high and low categories according to their urbanization index, population size and mortality rate (see main text for details).

The multistage stratification process included three steps: (i) counties and districts in each province were divided into two strata based on the median urbanization index for each province (i.e. high or low urbanization); (ii) counties and districts with a high or low urbanization index were further subdivided into two strata according to the median population size in each of the two urbanization strata in each province (i.e. high or low population size); and (iii) counties and districts in these four strata (i.e. two urbanization strata  × two population-size strata) were subdivided into two further strata using the median total mortality rate in each of these four strata in each province. This process yielded eight strata in each province (Fig. 4) and a total of 248 strata (i.e. 8 × 31 provinces) across the whole country.

### Ensuring representativeness

After stratification, counties and districts in each province were selected as surveillance points using inclusion criteria and principles developed in consultation with health administrators and disease surveillance experts. First, the number of selected counties and districts in each stratum of each province should be approximately *n*/8, where *n* is the number of counties and districts in each province included in the new surveillance points system (Table 3). Second, the existing 161 surveillance points in the current disease surveillance points system were considered as a priority for inclusion in the national mortality surveillance system. Then, other counties and districts with experience in mortality surveillance were also considered for inclusion, as far as possible. The vital registration system sites were given a lower priority during the selection process than disease surveillance points system sites because the vital registration system did not provide continuous, longitudinal, mortality data and the sites did not all have the same quality control measures in place. In addition, counties and districts in which local staff expressed a strong desire to participate and where there was demonstrable local government support were also considered. Finally, the counties and districts selected had to be evenly distributed across different geographical areas with different characteristics and all prefecture-level cities had to be included.

At each stage of the selection process, candidate surveillance points were evaluated to determine how representative they were of each province. Reselection and re-evaluation were repeated until the final population sample was considered sufficiently representative of the province. We employed an iterative process to ensure the representativeness of the candidate surveillance points (Fig. 1). First, the parameters used to evaluate representativeness were similar to those used in the two previous adjustments to the Disease Surveillance Points system:^8^^,^^9^ (i) the urbanization index; (ii)the ratio of the size of the population aged 65 years or more to the size of the total population; (iii)the ratio of the size of the population younger than 15 years to the size of the total population; and (iv) the crude mortality rate. Second, there had to be no significant difference between the value of a given parameter in the sample population and the corresponding value for the whole province, as indicated by a statistical test with the threshold of an *α*-level greater than 0.05. For variables that met the conditions for parametric tests, *t* tests were performed on log-transformed variables; otherwise, non-parametric tests were used. Although we aimed to select a similar number of counties or districts in each stratum of each province, this was not always possible. Because of the inclusion criteria and the variation in population size between counties and districts, inevitably the counties and districts in a province did not all have the same probability of being selected. Consequently, during the statistical evaluation of representativeness, we weighted each selected county and district according to its population.^12^^–^^14^

### Final surveillance points

After several rounds of representativeness evaluation and adjustment, we found that there was no significant difference in parameter values between the counties and districts chosen as surveillance points in each province and the entire province for all provinces in the national mortality surveillance system (Table 4). In total, 605 surveillance points were selected across China (Fig. 2): the number of counties and districts selected in each province varied from 7 to 36. Three of the 161 former sites in the disease surveillance points system were excluded because of poor data quality and limited local government support and capacity. Of the existing 319 vital registration system sites, 113 were retained in the national mortality surveillance system.

**Table 4:** Representativeness^a^ of points in the national mortality surveillance system, by province, China, 2013

Province	*P^b^*			
	Urbanization index^c^	Ratio ≥ 65 years^d^	Ratio < 15 years^e^	Mortality rate^f^
Anhui	0.69	0.17	0.35	0.36
Beijing	0.64	0.50	0.45	0.30
Chongqing	0.29	0.67	0.17	0.63
Fujian	0.52	0.88	0.42	0.75
Gansu	0.06	0.77	0.17 ^g^	0.31
Guangdong	0.83	0.94	0.21	0.41
Guangxi	0.42	0.80	0.55	0.44
Guizhou	0.23	0.69	0.39	0.43
Hainan	0.88	0.58	0.08 ^g^	0.74
Hebei	0.32	0.21	0.32	0.62
Heilongjiang	0.60	0.99	0.38	0.34
Henan	0.06	0.21	0.40	0.80
Hubei	0.72	0.66	0.19	0.43
Hunan	0.79	0.78	0.43	0.69
Inner Mongolia	0.29	0.17	0.13	0.70
Jiangsu	0.32	0.23	0.24	0.06
Jiangxi	0.67 ^g^	0.27	0.07 ^g^	0.55
Jilin	0.59	0.80	0.67	0.52
Liaoning	0.11	0.83	0.93	0.26^ g^
Ningxia	0.86	0.96	0.94	0.29
Qinghai	0.35	0.35	0.14	0.64
Shaanxi	0.32	0.78	0.46	0.62
Shandong	0.38	0.56	0.07	0.70
Shanghai	0.13	0.21	0.70	0.63
Shanxi	0.46	0.49	0.86	0.17
Sichuan	0.18	0.73	0.11	0.62
Tianjin	0.29	0.16	0.99	0.18
Tibet	0.10	0.15 ^g^	0.15 ^g^	0.11
Xinjiang	0.26	0.84	0.50	0.84
Yunnan	0.91	0.88	0.08	0.11
Zhejiang	1.00	0.65	0.45	0.13

^a^ Whether or not the population covered by the selected surveillance points in a province was representative of the population of the whole province was determined using the four parameters listed in the table.

^b^ We used either *t*-test or the Wilcoxon signed-rank test to calculate if there was significant difference between the parameter value across the surveillance points in a province and the corresponding value for the whole province.

^c^ Represents the fraction of the population residing in an urban area.

^d ^Ratio of the population aged 65 years or more to the total population.

^e ^Ratio of the population younger than 15 years to the total population.

^f ^Total number of deaths per 1000 people per year.

^g^ A non-parametric test was performed for this parameter.

In 2013, the national mortality surveillance system covered 323.8 million people. At the provincial level, the sample population ranged from 753 557 in Tibet to 25 919 659 in Guangdong. In five provinces (Hainan, Ningxia, Qinghai, Tibet and Xinjiang), the surveillance population was less than 5 million. The population covered by the surveillance points as a proportion of the total population in each province ranged from 14.5% in Shaanxi to 54.2% in Ningxia; it was over 20% in each of the five provinces with a surveillance population less than 5 million (Table 5).

**Table 5:** National mortality surveillance system surveillance population, by province, China, 2010

Province	Total population^a^	Surveillance population^a^	Proportion of total population (%)
Anhui	59 500 468	14 580 012	24.5
Beijing	19 612 368	6 605 681	33.7
Chongqing	28 846 170	8 167 594	28.3
Fujian	36 894 217	10 146 129	27.5
Gansu	25 575 263	7 803 139	30.5
Guangdong	104 320 459	25 919 659	24.8
Guangxi	46 023 761	9 025 031	19.6
Guizhou	34 748 556	8 997 602	25.9
Hainan	8 671 485	3 097 131	35.7
Hebei	71 854 210	13 896 574	19.3
Heilongjiang	38 313 991	10 069 900	26.3
Henan	94 029 939	21 785 954	23.2
Hubei	57 237 727	12 513 533	21.9
Hunan	65 700 762	17 661 802	26.9
Inner Mongolia	24 706 291	5 840 040	23.6
Jiangsu	78 660 941	22 084 484	28.1
Jiangxi	44 567 797	8 141 229	18.3
Jilin	27 452 815	7 044 428	25.7
Liaoning	43 746 323	10 729 378	24.5
Ningxia	6 301 350	3 417 327	54.2
Qinghai	5 626 723	1 649 165	29.3
Shaanxi	37 327 379	5 424 499	14.5
Shandong	95 792 719	23 067 010	24.1
Shanghai	23 019 196	8 055 902	35.0
Shanxi	35 712 101	7 222 517	20.2
Sichuan	80 417 528	18 066 478	22.5
Tianjin	12 938 693	5 548 441	42.9
Tibet	3 002 165	753 557	25.1
Xinjiang	21 815 815	4 532 804	20.8
Yunnan	45 966 766	9 269 827	20.2
Zhejiang	54 426 891	12 656 460	23.3
**Total**	**1 332 810 869**	**323 773 287**	**24.3**

^a^ Population data are from the 2010 national census.^11^

In 2014, the budget allocated by central government to run the national mortality surveillance system included two types of cost: (i) the cost of basic death registration procedures (i.e. collection, registration, reporting, quality control, supervision and training – 6444 United States dollars, US$, per surveillance point) and of periodic surveys of death underreporting (US$ 4833 per surveillance point); and (ii) the cost of work that varied with the estimated number of deaths, such as the printing, distribution and storage of registration cards (US$ 0.97 per death), the management and analysis of cause-of-death data (US$ 0.25 per death) and interdepartmental comparison and verification of data (US$ 0.25 per death).

## Discussion

In China, the ultimate aim is to establish a comprehensive vital registration and mortality surveillance system. However, in the interim, a sample-based mortality surveillance system^15^ is the only viable option for generating valid and reliable information on total and cause-specific mortality in the country. The establishment of the national mortality surveillance system with 605 surveillance points covering almost one quarter of the Chinese population is a highly significant step towards the goal of achieving the vital registration of all births and deaths across the country by 2020.

Perhaps the greatest advantage of the new system is that it will yield annual data on death rates and the causes of death for all provinces. The inclusion of most of the existing disease surveillance points system surveillance points in the new system ensures the continuity of mortality data from these points without affecting the national or regional representativeness of the data overall. The national mortality surveillance system is now the only mortality surveillance system in China covering all causes of death in people of all ages. Data from the 605 surveillance points will be reported at the time of death registration to the national CDC,^7^ which is responsible for the operation and maintenance of the information system. The National Health and Family Planning Commission is responsible for overall project management, policy-making and information dissemination.

One of the main objectives of the national mortality surveillance system is to reliably monitor specific causes of death at both national and provincial levels. Over the long term, surveillance data will become increasingly important for describing changes in mortality, for identifying emerging health-care priorities and for informing health policy development. Although knowing the extent to which the surveillance system reflects mortality patterns is useful for interpreting data, representativeness should not be the only factor considered when constructing a system and should not be overemphasized at the expense of practicality. Many leading epidemiologists have argued that representativeness is not imperative, especially when investigating causal inference or examining associations between diseases and their component causes.^16^^–^^18^ Our view is that building capacity and quality control should be the main priorities in implementing the new surveillance system in addition to ensuring representativeness.

The completeness of death registration and accurate coding of the cause of death and of identification of the underlying cause of death are key issues for any mortality surveillance system. Previous surveys of the disease surveillance points system found underreporting of 12 to 17% – the proportion was even higher among children younger than five years and in rural areas.^3^^,^^19^^,^^20^ Moreover, in 2012, a report indicated that 2.73% of causes of death in China were coded inaccurately.^21^ One of the main challenges for the new system is the high proportion of deaths occurring outside hospital. Traditional burial customs, including the desire to return to one’s place of original residence before death, mean that approximately 70% of deaths in rural areas (as much as 90% in some places) occur at home and medical records are limited or nonexistent. Second, staff at most new surveillance points lack relevant experience, especially with standardized workflow procedures and mechanisms for interdepartmental collaboration. Third, it may be difficult to recruit enough professional health workers, particularly at the local level. Fourth, there is an enormous difference in local capacity between the provinces due to large variations in economic development. To meet these challenges the integration and application of new automated methods for collecting information on the cause of death identified by verbal autopsy should be a priority.^22^^,^^23^ Also, uniform training materials should be used by national and provincial trainers to strengthen training; supervision and information-sharing should be enhanced and additional technical and financial support should be offered to underdeveloped provinces when necessary. In 2014, central government agencies, including the National Health and Family Planning Commission, the Ministry of Public Security and the Ministry of Civil Affairs, issued an updated official document aimed at strengthening death registration. However, appropriate legislation to ensure all deaths are registered and properly certified is also essential.

Reliable information on mortality and the cause of death is essential for the development of national and international health policy and of programmes for preventing and controlling disease and preventing injury. Data from the disease surveillance points and vital registration systems have been extensively used to assess the burden of disease both regionally and nationally in China and globally^9^^,^^10^^,^^24^^–^^26^ as well as for other research purposes.^27^^–^^29^ For the future, there are plans to use the national mortality surveillance system sample populations to carry out periodic national surveys of chronic disease, nutrition and injury. Electronic linkage of data is becoming easier in China and it may soon be possible to convert these periodic surveys into prospective cohort studies. The national mortality surveillance system will not only play a unique and critical role in providing health metrics for China but will also serve as an essential resource for evaluating health-care policy at provincial, national and international levels, particularly for the prevention and control of chronic diseases.
